# Role of CCCH-Type Zinc Finger Proteins in Human Adenovirus Infections

**DOI:** 10.3390/v12111322

**Published:** 2020-11-18

**Authors:** Zamaneh Hajikhezri, Mahmoud Darweesh, Göran Akusjärvi, Tanel Punga

**Affiliations:** 1 Department of Medical Biochemistry and Microbiology, Uppsala University, 75123 Uppsala, Sweden; Zamaneh.Hajikhezri@imbim.uu.se (Z.H.); Mahmoud.Darweesh@imbim.uu.se (M.D.); Goran.Akusjarvi@imbim.uu.se (G.A.); 2 Department of Microbiology and Immunology, Al-Azhr University, Assiut 11651, Egypt

**Keywords:** human adenovirus, zinc finger protein, CCCH-type, ZC3H11A, MKRN1, U2AF1

## Abstract

The zinc finger proteins make up a significant part of the proteome and perform a huge variety of functions in the cell. The CCCH-type zinc finger proteins have gained attention due to their unusual ability to interact with RNA and thereby control different steps of RNA metabolism. Since virus infections interfere with RNA metabolism, dynamic changes in the CCCH-type zinc finger proteins and virus replication are expected to happen. In the present review, we will discuss how three CCCH-type zinc finger proteins, ZC3H11A, MKRN1, and U2AF1, interfere with human adenovirus replication. We will summarize the functions of these three cellular proteins and focus on their potential pro- or anti-viral activities during a lytic human adenovirus infection.

## 1. Zinc Finger Proteins

Zinc finger proteins are a big family of proteins with characteristic zinc finger (ZnF) domains present in the protein sequence. The ZnF domains consists of various ZnF motifs, which are short 30–100 amino acid sequences, coordinating zinc ions (Zn^2+^). Other metals, such as cobalt, copper, nickel, and cadmium ions, can also associate with the ZnF motifs and compete with zinc ions when binding to the ZnF motifs [1].

The *Xenopus laevis* transcription factor TFIIIA has played a key role in understanding ZnF structures. This essential protein contains nine consecutive, 30 amino acid long sequence motifs, which fold around zinc ions and form a structure that visually resembles a “finger”, hence the name zinc finger [2,3]. The cysteine (C) and histidine (H) residues are important for the ZnF motifs and coordinate the zinc ions and thereby stabilize the ZnF structure. A classic example here is the TFIIIA ZnF motif, where the zinc ion is maintained by two cysteine and two histidine residues (C_2_H_2_). Substitutions of the zinc coordinating histidine residues within the ZnF motifs inhibit TFIIIA function as a transcription factor [4,5]. Not all zinc coordinating histidine substitutions within individual ZnF motifs affect TFIIIA function, indicating that ZnF motifs are not functionally equivalent [4,5].

The last four decades have revealed that the ZnF containing proteins are very abundant in eukaryotic cells. It has been estimated that at least 3% of all human genes encode the ZnF proteins with abundant C_2_H_2_ and C_4_ motifs [6]. Even though many of the ZnF proteins contain the classical C_2_H_2_ motif, there are still numerous proteins with atypical ZnF motifs [7,8]. These non-classical ZnFs have slightly different cysteine and histidine combinations, which can be used to discriminate between different ZnF types. For example, in the CCCH-type ZnF proteins, a zinc ion is coordinated by three cysteines and a single histidine (C-x-C-x-C-x-H), whereas in the CCHC-type ZnF, the two cysteines are followed by the single histidine and cysteine (C-x-C-x-H-x-C) residues to coordinate the zinc ion. Currently, more than 40 different types of ZnF proteins are annotated in the UniProt Knowledgebase (www.uniprot.org), with the C_2_H_2_-, CCHC-, PHD-, and RING-types being the most common types. As the present review deals only with the CCCH-type ZnF proteins, we recommend additional excellent reviews about different ZnF proteins for interested readers [9,10].

The ZnF proteins make up a significant part of the human proteome, therefore it is not surprising that they carry out very diverse biological functions. Truly, various ZnF proteins have been shown to be involved in chromatin remodeling, transcription activation/repression, DNA repair, regulation of apoptosis, folding and assembly of proteins, stress response, cell proliferation, and differentiation [9,10,11]. Many of the C_2_H_2_-type ZnFs are characterized as sequence-specific DNA-binding proteins acting as transcription activators or repressors [12,13]. The ZnF proteins can also bind to RNA, as first described for the TFIIIA protein which associates with double-stranded regions in the 5S ribosomal RNA [2]. The TFIIIA protein is an example of the ZnF protein that is able to bind to both DNA and RNA, with different zinc fingers engaged in DNA and RNA binding [14]. Furthermore, the ZnF domains appear to be multifunctional since in addition to binding to nucleic acids, they can also mediate multiple protein-protein interactions and recognize a variety of post-translational modification [15,16].

## 2. CCCH-Type Zinc Finger Proteins 

Although the ZnF proteins are generally thought of as DNA-binding proteins, the CCCH-type (C_3_H_1_) ZnF proteins, where a zinc ion is coordinated by three cysteines and a single histidine, commonly function as RNA-binding proteins with an important function in RNA metabolism. Genome-wide surveys have revealed that there are currently 57 different CCCH-type ZnF proteins in humans, with about 1/3 lacking a known function [17,18]. Of the characterized CCCH-type ZnF proteins, most are involved in different steps in RNA metabolism. This includes pre-mRNA splicing (U2AF1, see below), polyadenylation (ZC3H3), mRNA export (ZC3H11A, see below), translation (ZC3H1), and mRNA stability (RC3H1). In addition, some CCCH-type ZnF proteins control protein stability by ubiquitination (MKRN1, see below), act as transcriptional repressor proteins (ZC3H8), and adjust RNA structures (ZC3H12A) [17,19]. 

Many of the CCCH-type ZnF proteins are involved in inflammation and immunity, immune homeostasis, and immune cell maturation [17,19]. This can be partially explained by specific interaction of some of the CCCH-type ZnF proteins with cytokine mRNAs and their ability to carry out post-transcriptional processing of the targeted mRNAs [19]. Mechanistically, the best characterized are the ZFP36 (also known as tristetraproline (TTP)), RC3H1 (also known as Roquin 1), and ZC3H12A (also known as Regnase-1/MCPIPI1) proteins. 

These ZnF proteins seem to control cytokine mRNA stability, although by different mechanisms. For example, the ZFP36 protein binds to the AU-rich elements at the 3′ untranslated region (3′ UTR) of TNF-α and IL-6 mRNAs [20,21]. RNA-bound ZFP36 can recruit proteins involved in mRNA decapping (e.g., Edc3) and deadenylation (e.g., Not1), which will initiate mRNA degradation [22]. In addition, ZFP36 is an essential regulator of the NLRP3 inflammasome activity since it destabilizes both NLRP3 and IL-1β mRNAs [23]. The RC3H1 protein binds to mRNA via its ROQ and CCCH-type ZnF domains [24]. The RC3H1 protein causes TNF-α mRNA degradation by binding to the constitutive decay element (CDE) present at the 3′ UTR of TNF-α mRNA and recruits mRNA deadenylase complex Ccr4-Caf1-Not to stimulate TNF-α mRNA degradation [25]. Likewise, the ZC3H12A protein binds to the 3′ UTR of the IL-6 mRNA. Since the ZC3H12A protein has an intrinsic ribonuclease activity, the targeted mRNA is cleaved and degraded by this protein [26]. Notably, the ZC3H12A and RC3H1 proteins can collaborate with each other by targeting overlapping sets of mRNAs via a common stem-loop structure [27]. Even though these examples are mainly about TNF-α and IL-6 mRNAs, the high-throughput RNA-binding experiments have revealed that many more inflammation- and immune-related mRNAs are targeted by the ZFP36, RC3H1, and ZC3H12A proteins [27,28].

Remarkably, out of 57 CCCH-type ZnF proteins, at least nine proteins have been assigned either an anti- or pro-viral function [17,29,30]. Here, most attention has been focused on the zinc finger anti-viral protein (ZAP, also known as ZC3H2, ZC3HAV1, PARP13), which is considered an anti-viral protein targeting a number of RNA viruses. In fact, ZAP has been shown to restrict the growth of retroviruses, alphaviruses, filoviruses, influenza virus, and SARS-CoV-2 [31,32,33,34,35,36]. Even though ZAP is regarded as a broad-range RNA virus inhibitor, several RNA viruses (e.g., vesicular stomatitis virus, poliovirus, yellow fever virus, dengue virus, Zika virus) are still resistant to ZAP’s anti-viral activity [31,37]. The N-terminus of the ZAP contains four CCCH-type ZnF motifs. Recent studies have shown that ZAP can selectively bind to CG-rich RNAs, with its highly basic second ZnF motif being essential for binding to the CG dinucleotides [36,38,39]. Furthermore, ZAP interacts directly with CG-rich regions in the HIV-1 RNA. As a consequence of this, unspliced HIV-1 RNAs are selectively depleted in the cytoplasm, which results in an inhibition of virus replication and virus progeny formation [36]. In addition to a direct RNA binding, the ZAP protein makes several protein-protein contacts with factors involved in mRNA translation and RNA processing. For example, ZAP interaction with translation initiation factors eIF4G and eIF4A, which are part of the eIF4F cap recognition complex, inhibits translation of viral mRNA [40]. ZAP also associates with several components of the RNA 5′-3′ and 3′-5′ degradation complexes, which may regulate the stability of viral RNAs [41]. 

## 3. Human Adenoviruses and CCCH-Type Zinc Finger Proteins 

Human adenoviruses (HAdVs) are widespread pathogens causing ocular, respiratory, and gastrointestinal diseases, with more than 100 different virus types identified so far [42] (http://hadvwg.gmu.edu). Despite such a variety, the majority of studies have been focused on common HAdV types 2 and 5 (HAdV-2 and HAdV-5). To accomplish its replication, HAdV encode multiple proteins and non-coding RNAs that interfere with the normal function of cellular proteins, thereby reprogramming the host cell transcriptome and proteome to more effectively produce new virus progeny [43,44,45]. Analogously to other DNA viruses, the HAdV gene expression pattern is subdivided into an early and a late phase based on the expression timing of the respective genes. Generally, HAdV early genes (e.g., E1A, E1B, E2, E3) encode viral proteins involved in suppression of the host cell response, deregulation of the cell cycle, and initiation of virus DNA replication. The majority of the late phase-specific genes are encoded from a single transcription unit, the so-called major late transcription unit (MLTU), which becomes highly active after initiation of virus DNA replication. Most of the HAdV late genes encode for structural proteins of the virion, such as hexon, penton, fiber, and protein VII, which are needed to assemble infectious virus particles [43]. Typically, HAdV infections cause cell lysis (i.e., lytic infection), even though some HAdV types can also establish long-term persistent infections in T and B cells [46,47].

HAdVs seem to encode only one known ZnF protein, the E1A-289R protein, which contains the C_4_-type ZnF motif in the conserved region 3 (CR3) part of the protein [48]. The E1A-289R protein is an intrinsically disordered protein that functions as a hub, mediating primary interactions with more than 50 cellular proteins. The E1A ZnF is believed not to make direct RNA contacts, but instead mediates protein-protein interactions with different cellular transcription factors [49].

**Figure 1:**
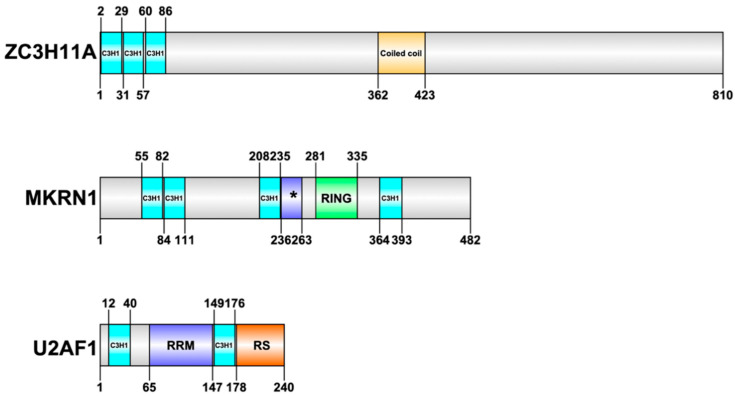
Structural overview of three CCCH-type zinc finger proteins: ZC3H11A (O75152), MKRN1 (Q9UHC7), and U2AF1 (Q01081). The domain labelling is based on Uniprot annotation (www.uniprot.org). Abbreviations: C3H1; CCCH-type zinc finger motif, asterisk (*); makorin-type Cys-His region, RRM; RNA recognition motif, RING; RING finger domain, Coiled coil; Coiled coil domain, RS; Arg/Ser-rich domain. Uniprot identifier is indicated in the parentheses.

Nevertheless, HAdVs interfere with several cellular CCCH-type ZnF proteins during the virus lifecycle [30,50,51,52]. Here, we will review our current knowledge of the function of three cellular CCCH-type ZnF proteins (ZC3H11A, MKRN1, U2AF1) that have been studied in details during HAdV infection (Figure 1).

### 3.1. ZC3H11A as a Pro-Viral Factor Promoting HAdV-5 mRNA Export

ZC3H11A is a CCCH-type ZnF protein with three ZnF motifs present at the beginning of the N-terminus of the protein (Figure 1). Structural prediction programs suggest that most of the protein, excluding the ZnF and coiled coil domains, is to a large extent intrinsically disordered. For a long time, ZC3H11A functions remained elusive. The first indication of a potential function came from two large-scale proteomics studies demonstrating that ZC3H11A is one of the components of the Transcription-Export (TREX) complex [53,54]. TREX is a multiprotein complex that is conserved from yeast to humans and serves a key function in nuclear export of mRNAs [55]. The TREX complex interacts with the mRNA capping complex and the exon junction complex (EJC), thereby integrating TREX to the nuclear export pathway of capped and spliced mRNAs [53]. This also explains why the ZC3H11A protein is found to interact with various mRNA capping and splicing factors [56,57]. Despite the fact that the ZC3H11A protein co-purifies with the individual members of the TREX complex, its exact role in the complex still remains, to a large extent, enigmatic. Interestingly, elimination of the ZC3H11A protein with an siRNA approach increased accumulation of polyadenylated mRNA in the cell nucleus, suggesting a direct involvement of ZC3H11A in mRNA export [53].

It has recently been shown that CRISPR/Cas9 knock-out of ZC3H11A in HeLa cells (hereafter referred to as ZC3H11A^KO^ cells) does not impair HeLa cell growth under normal conditions [30]. However, ZC3H11A^KO^ cells showed a retarded growth after heat-shock, suggesting that ZC3H11A is a stress-induced protein protecting cells against the harmful effects of stress. Similarly, a virus infection could also be regarded as a stress-inducer. In fact, in ZC3H11A^KO^ cells, multiple nuclear-replicating viruses (HIV-1, HAdV-5, influenza virus (IAV), and herpes simplex virus (HSV-1) were stalled in their growth, whereas cytoplasmic replicating viruses (vaccinia virus (VACV) and Semliki Forest virus (SFV)) were not [30]. These data indicate that nuclear replicating viruses have evolved to take advantage of the stress-induced ZC3H11A protein to facilitate virus growth, something that cytoplasmic replicating viruses cannot use since these viruses are not, to the same extent, dependent on the nuclear TREX export machinery for virus growth.

**Figure 2:**
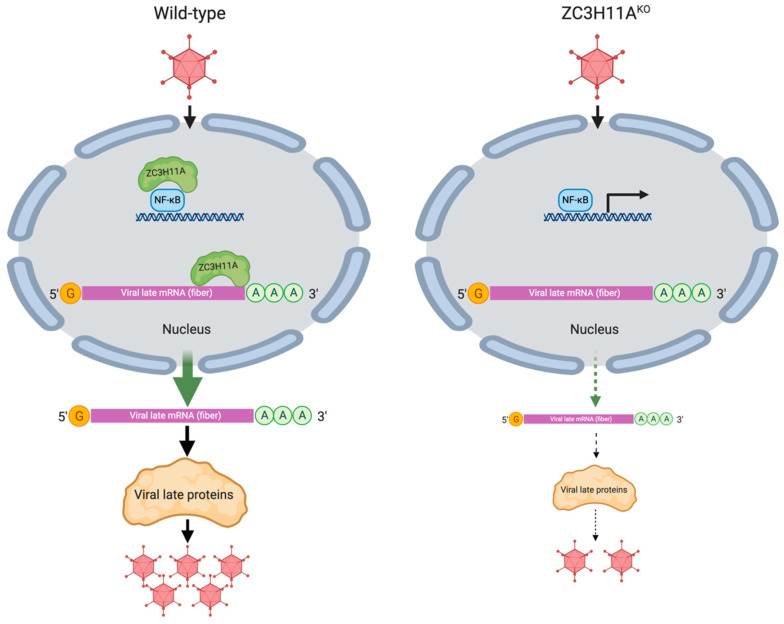
Proposed model of ZC3H11A pro-viral role in human adenovirus type 5 (HAdV-5) infected cells. Increased accumulation of the ZC3H11A protein promotes selective virus late mRNA (e.g., fiber mRNA) nuclear export during late phase of HAdV-5 infection (Wild-type). Lack of the ZC3H11A protein (ZC3H11A^KO^) reduces HAdV-5 late mRNA nuclear export, virus late protein synthesis, and formation of infectious virus progeny. ZC3H11A interference with the NF-κB signaling pathway prevents expression of pro-inflammatory genes, a block that is relieved in ZC3H11A^KO^ cells. Figure created with Biorender.com.

Furthermore, it was shown that ZC3H11A, via its three ZnF motifs (Figure 1), binds to short purine-rich sequences in cellular and HAdV-5 RNAs [30]. However, the binding specificity for ZC3H11A changed with a more complex binding motif identified in HAdV-5-infected cells. This change in specificity was also observed by a significant change in the cellular mRNAs that were targeted by ZC3H11A in HAdV-5-infected cells compared to uninfected cells. In general, the cellular mRNAs targeted by the ZC3H11A protein are involved in mRNA metabolic processes, pre-mRNA splicing, and the cellular response to stress. In ZC3H11A^KO^ cells, the viral fiber mRNA was retained in the nucleus, lending support to the hypothesis that the ZC3H11A protein facilitates viral mRNA export. In line with the defect in virus mRNA export, the expression of the viral late proteins was also affected in ZC3H11A^KO^ cells (Figure 2). However, it is noteworthy that accumulation of all late structural proteins except the hexon protein was drastically reduced in ZC3H11A^KO^ cells, suggesting the interesting possibility that the hexon mRNA may use an alternative export pathway [30]. 

The nuclear localization of ZC3H11A changed during a HAdV-5 infection [30]. In uninfected cells, ZC3H11A accumulates in nuclear speckles, which are storage sites for RNA processing factors [58]. In HAdV-5-infected cells, the localization of ZC3H11A, along with cellular splicing factor SRSF2, changed dramatically to form foci at the so-called viral replication centers where viral DNA replication, transcription, and RNA processing takes place [59]. Interestingly, the ZC3H11A protein levels have been shown to increase in both HAdV-2 and HAdV-5 infected cells, specifically during the late phase of infection [30,60]. This is an unusual phenomenon since a HAdV infection in general hinders translation of most cellular mRNAs during the late phase of infection [60]. The increase in ZC3H11A protein accumulation was not accompanied by a similar increase in ZC3H11A mRNA expression, suggesting that the increase in protein expression is regulated at the level of translation or via post-translational mechanisms [30].

A high-throughput RNA sequencing experiment in ZC3H11A^KO^ cells revealed a significant up-regulation of innate immune related mRNAs, especially those downstream of the NF-κB and interferon type 1 signaling pathways [61]. Hence, ZC3H11A appears to be a factor involved in negative regulation of the NF-κB signaling pathway (Figure 2).

The ZC3H11A protein has been found to colocalize with the splicing factor SRSF2 and the m6A (N6-adenosine methylation) reader protein YTHDC1 in nuclear speckles [62]. Further, m6A is the most abundant RNA modification in eukaryotes and is regulated by the: “writer” proteins, which deposit the methyl group onto mRNAs; “reader” proteins, which define the fate of the m6A modified mRNAs; and “eraser” proteins, which remove the m6A signal from mRNA [63]. Interestingly, a recent study found that the m6A reader protein YTHDC1 was redistributed into the viral replication centers in HAdV-5-infected cells [64]. The same study also showed that elimination of the YTHDC1 protein significantly reduced HAdV-5 late fiber mRNA splicing. Taken together, a lack of the ZC3H11A or YTHDC1 proteins seem to specifically affect fiber mRNA biogenesis. Since mRNA splicing is coupled to export, it is possible that ZC3H11A may export m6A modified and YTHDC1-bound viral mRNAs, such as fiber mRNA, to the cytoplasm.

Abnormal ZC3H11A expression or protein interactions have been found in several human diseases and cancers. For example, the expression level of ZC3H11A is significantly higher in breast cancer tissues than in normal tissue [65,66]. It has also been shown that there is significant overexpression of ZC3H11A in mutant KRAS lung adenocarcinomas [67]. KRAS is a proto-oncogene and its mutations are the most common molecular alteration found in non-small cell lung cancers, hence representing one of the predictors of poor prognosis in this cancer [68]. Further, it has been reported that ZC3H11A associates with a mutant version of the nuclear matrix protein, Matrin-3, which has been found in patients with amyotrophic lateral sclerosis (ALS) [69]. This study showed that ALS-linked mutations increase Matrin-3 co-localization with the TREX complex components, which may explain the nuclear mRNA export defects in ALS patients [69].

### 3.2. MKRN1 as a Potential Anti-Viral Factor in HAdV-5 Infection

Makorin ring finger protein 1 (MKRN1) is another CCCH-type ZnF protein shown to be engaged in the HAdV-5 lifecycle [50]. The MKRN1 protein consists of four C_3_H_1_-type motifs and one C_3_HC_4_-type RING finger domain (Figure 1). There are three MKRN proteins (MKRN1, MKRN2, and MKRN3) identified within the human MKRN protein family, with MKRN1 as an ancestral gene of the family [70]. 

In contrast to ZC3H11A, the MKRN1 protein has been relatively well characterized. The MKRN1 protein functions as an E3 ubiquitin ligase since it has a functional RING finger domain, one of the fundamental features of the E3 ubiquitin ligases [71]. MKRN1 mediates ubiquitination of several substrate proteins, including Fas-associated protein with death domain (FADD), human telomerase reverse transcriptase (hTERT), p14ARF, p21, p53, peroxisome-proliferator-activated receptor γ (PPARγ), and AMP-activated protein kinase (AMPK) [29,71,72,73,74,75]. Since MKRN1 induces p53, p21, and p14ARF degradation, it is thought to be an important regulator of the cell cycle and apoptosis [76]. In addition to the E3 ubiquitin ligase activity, the MRKN1 protein is an RNA-binding protein. Original findings by Cassar and co-workers showed that MKRN1 is a stress-granule-resident protein that is associated with mRNAs encoding proteins that function during cellular stress [77]. A more recent study has shown that MKRN1 is involved in ribosome-associated quality control of prematurely polyadenylated mRNAs [78]. Based on this study, MKRN1 positioning upstream of mRNA poly(A) tails and MKRN1-mediated degradation of cytoplasmic poly(A)-binding protein (PABPC1) ensures ribosome stalling upstream of the poly(A) sequences. As proposed by the authors, this mechanism blocks translation of erroneous proteins from prematurely polyadenylated mRNAs [78]. 

Regarding a HAdV infection, the MKRN1 protein was identified as a binding partner of the ubiquitous HAdV histone-like protein VII (pVII) [50]. Surprisingly, this interaction was shown to cause self-ubiquitination of the MKRN1 protein. Further, the MKRN1 protein is efficiently degraded by the host cell proteasome, which overlaps with *de novo* accumulation of the viral pVII protein during the late phase of virus infection. Additional experiments have shown that transient overexpression of the MKRN1 protein reduces accumulation of the viral capsid proteins, which coincides with a decreased formation of infectious virus particles (R. Inturi, personal communication). In the same study, it was also shown that the amount of MKRN1 protein was remarkably reduced in measles virus (MV) and vesicular stomatitis virus (VSV) infected cells, although the exact molecular mechanisms behind these observations were not revealed [50].

Taken together, this study [50] suggests that MKRN1 may function as a potential anti-viral factor in HAdV-5 infection and that the viral pVII protein induces the MKRN1 protein self-ubiquitination and proteasomal degradation.

MKRN1 could be considered as a potential widespread anti-viral protein since it interferes with additional virus infections. For example, MKRN1 can specifically induce ubiquitination and proteasomal degradation of the West Nile virus (WNV) capsid protein. As a consequence of that MKRN1 inhibits WNV replication and protects cell against WNV-induced cell death [29]. Similarly, porcine MKRN1 (pMKRN1) has been shown to modulate porcine circovirus type 2 (PCV2) replication. The pMKRN1 can induce ubiquitination and proteasomal degradation of the PCV2 capsid protein and thereby reduces virus progeny production [79].

### 3.3. U2AF1 as a Potentially Dispensible Factor in HAdV Late Alternative RNA Splicing

U2AF (U2 small nuclear ribonucleoprotein (snRNP) auxiliary factor) is a splicing factor required for the stable recruitment of U2 snRNP to the 3′ splice site in a pre-mRNA during the early stages of spliceosome assembly [80]. U2AF is a heterodimer consisting of a 65- (U2AF2) and 35-kDa (U2AF1) subunits. Both U2AF subunits are RNA-binding proteins. U2AF1 contains a putative RNA recognition motif (RRM) flanked by two CCCH-type ZnF motifs (Figure 1). This putative RRM does not mediate RNA contacts and instead functions as the interaction surface when U2AF1 associates to U2AF2 [81]. The CCCH-type ZnFs appear to mediate the RNA contacts in U2AF1. U2AF2 contains three RRM motifs and an N-terminal RS domain, which is the signature structure for RNA splicing factors. Similarly to the RRM-like motif in U2AF1, the first RRM in U2AF2 does not bind RNA and instead mediates protein-protein contact with splicing factor SF1, which is involved in 3′ splice site recognition. In spliceosome assembly, U2AF2 specifically binds to the pyrimidine tract close to the 3′ splice site, whereas U2AF1 makes contact with the conserved 3′ splice site AG dinucleotide [82,83]. Introns with weak pyrimidine tracts that bind U2AF2 inefficiently require the U2AF1 subunit interaction with the 3′ splice site AG to be functional, so-called AG-dependent introns. In contrast, 3′ splice sites with strong pyrimidine tracts that bind U2AF2 efficiently can splice without the contribution of U2AF1 interaction with the 3′ splice site AG, so-called AG-independent introns [84].

The significance of U2AF in HAdV splicing has been studied in some detail. U2AF, which is an essential splicing factor, is normally localized to nuclear speckles in the G1 phase of the cell cycle. These sites are believed to be storage sites for inactive RNA processing factors. After a HAdV infection, U2AF becomes redistributed from the nuclear speckles to so-called viral replication centers, which are sites for active viral transcription and RNA processing. The U2AF2 RS domain is essential for the recruitment of U2AF to the viral replication centers [85]. 

The requirement of U2AF for splicing in the HAdV system has been most extensively studied in vitro, using the major late transcription unit (MLTU) L1 family of mRNAs as a model substrate. In this unit, a common 5′ splice site is spliced to two alternative 3′ splice sites, resulting in the formation of the so-called 52,55K or IIIa mRNAs [86]. The 52,55K 3′ splice site has a consensus type of long polypyrimidine tract that binds U2AF2 efficiently, whereas the IIIa 3′ splice site has a weak sequence context with a short pyrimidine tract. During virus infection, a temporal shift in 3′ splice site choice occurs, resulting in the activation of the IIIa splice site in late virus-infected cells [86]. Previous work has demonstrated that activation of IIIa mRNA splicing is controlled by the 28-nucleotide long sequence element coinciding with the IIIa 3′ splice site; the so-called virus infection-dependent splicing enhancer (the 3VDE) [87]. U2AF is an essential splicing factor for 52,55K splicing both in nuclear extracts prepared from uninfected and HAdV-infected cells. The major late first intron, which like the 52,55K 3′ splice site has an extended polypyrimidine tract, is as expected, U2AF1 independent since U2AF2 binds efficiently to the extended polypyrimidine tract. The weak IIIa 3′ splice site requires U2AF1 for activity in nuclear extracts (NE) prepared from uninfected cells [51]. This result was anticipated since U2AF1 binding to the 3′ splice site AG is needed for splicing of weak introns. In contrast, U2AF1 appears to be completely dispensable for IIIa splicing in nuclear extracts prepared from HAdV late infected cell (Ad-NE) [51]. Thus, the IIIa 3′ splice site is transformed from a U2AF1-dependent to a U2AF1-independent intron in Ad-NE. In fact, the experimental data suggest that the 3VDE operates through a novel mechanism that appears to be completely U2AF-independent in HAdV-5 infected cells [51]. 

Collectively, available data suggest that the cellular spliceosomal machinery undergoes a drastic change in specificity during a HAdV infection. Clearly, the change in U2AF1 requirement at the late stage of IIIa pre-mRNA splicing is a first signature of this reformation.

## 4. Conclusions and Future Perspectives

The CCCH-type ZnF proteins have emerged as the essential regulators of RNA metabolism. Particularly, their regulatory roles in different virus infections have put them into the spotlight as possible targets for anti-viral therapies. Even if most HAdV infections are self-limiting, fatal infections can occur in immuno-compromised hosts and occasionally in healthy children and adults infected with particular HAdV types (e.g., HAdV-7) [88,89]. The observations that the enigmatic ZC3H11A protein promotes HAdV-5 late mRNA export and that a lack of this protein severely inhibits HAdV-5, HIV-1, IAV, and HSV-1 growth point towards the specific role of this protein in different virus lifecycles [30]. Hence, interference with the ZC3H11A protein functions may be considered as a potential therapeutic intervention point. One possibility here is to use metal-based compounds which can compete with zinc ions for binding to the ZnF motifs. Truly, zinc ion replacement with gold, platinum, cobalt, and selenium complexes can disrupt ZnF protein binding to nucleic acids [90]. Further, cisplatin, a well-known platinum-based anti-cancer drug, can selectively bind to and cause structural perturbation of some ZnF motifs [91]. It remains to be tested whether any of the metal-based compounds can alter the biological functions of the ZC3H11A protein. 

There are still several basic questions that need to be answered about the ZC3H11A protein. For example, how does the ZC3H11A ZnF domain interact with RNA and what is the contribution of individual ZnF motifs in this process? This question will apply also to other CCCH-type ZnF proteins, such as MKRN1, as only a few of them (e.g., ZAP) have established crystal structures to explain their RNA binding specificities [38,39]. Further, it will be of interest to understand how different post-translational modifications control ZC3H11A functions in the infected cells. In line with that, the ZC3H11A protein is heavily sumoylated in heat shock-treated cells [92]. However, the contribution of this modification in different virus infections has not yet been investigated. 

In contrast to MKRN1, which establishes a firm complex with the HAdV-5 pVII protein [50], nothing is known about ZC3H11A interference with viral proteins. During the late phase of infection, viral late mRNAs are efficiently exported, whereas the cellular mRNAs tend to accumulate in the nucleus [93]. This process is controlled by two viral proteins—E1B-55K and E4orf6 [93,94]. Since ZC3H11A is involved in mRNA export, it would be of interest to study if the ZC3H11A protein interacts with the E1B-55K/E4orf6 complex to achieve selective viral mRNA export during the late phase of infection. 

ZC3H11A appears to be a multifunctional protein (Figure 2). With this in mind, can we assign other biological functions to the ZC3H11A protein, in addition to its role in mRNA export and NF-κB signaling? Regarding its predominant nuclear localization [30], it is likely that the protein is also directly involved in different co- and post-transcriptional processes.

The finding that the L1-IIIa pre-mRNA splicing becomes U2AF1 independent at late times of infection opens up a possible model for how HAdV remodels the host RNA splicing machinery to selectively process the late viral pre-mRNAs. Thus, it is possible that much of MLTU alternative splicing, like the L1-IIIa pre-mRNA splicing, would work in the absence of U2AF1, creating an environment where host cell splicing, which to a large extent is U2AF1-dependent, would be shut off. Such a mechanism would be the equivalent to how HAdV selectively shuts off host cell translation late during infection [60]. Clearly, previous work needs to be expanded to also include an analysis of the global effect of U2AF1 depletion on early and late HAdV pre-mRNA splicing.

Taken together, we are confident that the coming years will present us with several exciting studies, revealing both the structural and functional characteristics of the CCCH-type ZnF proteins and their interplay with different virus infections.
